# Dunking Pancreaticojejunostomy With Braun’s Jejunojejunostomy: Perioperative Outcomes of a Hundred Pancreaticoduodenectomies

**DOI:** 10.7759/cureus.68988

**Published:** 2024-09-09

**Authors:** Sampanna Pandey, Narendra Maharjan, Sumita Pradhan, Ramesh S Bhandari

**Affiliations:** 1 Department of Surgical Gastroenterology, Tribhuvan University Teaching Hospital, Maharajgunj Medical Campus, Institute of Medicine, Kathmandu, NPL

**Keywords:** braun’s, complications, delayed gastric emptying, pancreaticoduodenectomy, pancreaticojejunostomy

## Abstract

Background

The optimal surgical technique for pancreaticojejunostomy (PJ) following pancreaticoduodenectomy (PD) is still debated. Dunking and duct-to-mucosa PJ are the most commonly adopted techniques. Incorporating Braun’s jejunojejunostomy (JJ) could reduce the incidence and severity of delayed gastric emptying (DGE). This retrospective descriptive study seeks to clarify the outcomes of dunking PJ with Braun’s JJ in 100 PD patients.

Methodology

We retrospectively reviewed 100 patients who underwent PDs in a single unit of the Department of Surgical Gastroenterology of Tribhuvan University Teaching Hospital from October 2012 to February 2023. Demographic and historical data, indications, procedure-related data, complications, and mortality data were collected and analyzed.

Results

The mean age was 50.96 ± 14.97 years, and 64 (64%) were males. The most common indication was ampullary carcinoma (53, 53%) followed by distal cholangiocarcinoma (18, 18%) and pancreatic ductal adenocarcinoma (7, 7%). Operative time was 5.83 ± 1.09 hours, intraoperative blood loss was 515 ± 194 mL, and the average time for PJ and Braun’s JJ was 22 ± 6 and 15 ± 3 minutes, respectively. Soft pancreas was encountered in 52 (52%) patients and clinically significant postoperative pancreatic fistula (Grade B, C) was seen in 23 (23%). Postpancreatectomy hemorrhage was seen in 21 (21%) patients (Grades A: 3, B: 12, C: 6). DGE (Grade B) occurred in two (2%) patients, and bile leak was observed in 4% of patients (Grades A: 1, B: 2, C: 1). Major complications (Clavien-Dindo ≥IIIA) occurred in 24%, and 11 patients died.

Conclusions

The dunking technique is easily adaptable, less time-consuming, and can be performed in the pancreas of any texture or duct size but is associated with an increased incidence of post-pancreatectomy hemorrhage. Incorporation of Braun’s anastomosis lowers DGE, allows early initiation of feeding, and reduces complication rates.

## Introduction

Pancreaticoduodenectomy (PD), one of the most complex intra-abdominal procedures, is widely utilized for malignant as well as benign ailments involving the pancreas and periampullary region [1]. Various methods of anastomosis between the pancreas and jejunum or stomach have been described. Pancreatico-enteric anastomosis is referred to as the Achilles heel of PD, and the ideal technique of reconstruction is debated [2]. Major complications of the procedure, ranging from 40-50%, are mostly associated with postoperative pancreatic fistula (POPF), which is directly related to the integrity of pancreaticojejunal (PJ) anastomosis [3]. Of the various techniques of PJ described in the literature, dunking, duct-to-mucosa, or a combination of these techniques are commonly utilized [4]. Various studies have shown no difference in POPF, delayed gastric emptying (DGE), overall morbidity, and mortality among these two techniques [5,6]. DGE is another debilitating complication, occurring in 30-52% of PDs. Braun’s jejunojejunostomy (JJ), which involves anastomosis of the afferent and efferent limbs distal to gastrojejunostomy (GJ), has been shown to decrease the rate and severity of DGE [7].

Although several studies have investigated the technique of PJ and GJ, none have examined the outcome of dunking PJ with Braun’s JJ. Thus, this retrospective descriptive study aimed to investigate the outcomes of patients undergoing PD with reconstruction with dunking PJ and Braun’s JJ.

The preliminary results of this paper were the subject of an abstract presented at the 15th International Conference of the Society of Surgeons of Nepal (XV ICSSN 2023), on December 2, 2023.

## Materials and methods

This retrospective descriptive study was conducted in a single unit of the Department of Surgical Gastroenterology at Tribhuvan University Teaching Hospital, Kathmandu, Nepal. All patients who had undergone PD from October 2012 to February 2023 were included in the study. Demographic and historical data, indications, procedure-related data, complications, and mortality data were collected. Data are presented as numbers, mean ± SD, and percentages where appropriate using SPSS version 26 (IBM Corp., Armonk, NY, USA).

Method of dunking pancreaticojejunostomy

**Figure 1:**
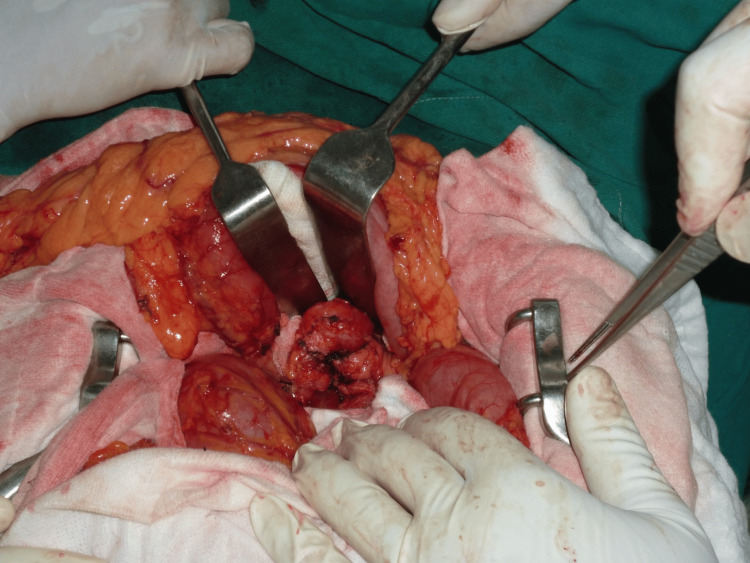
Mobilization of the pancreatic remnant.

**Figure 2:**
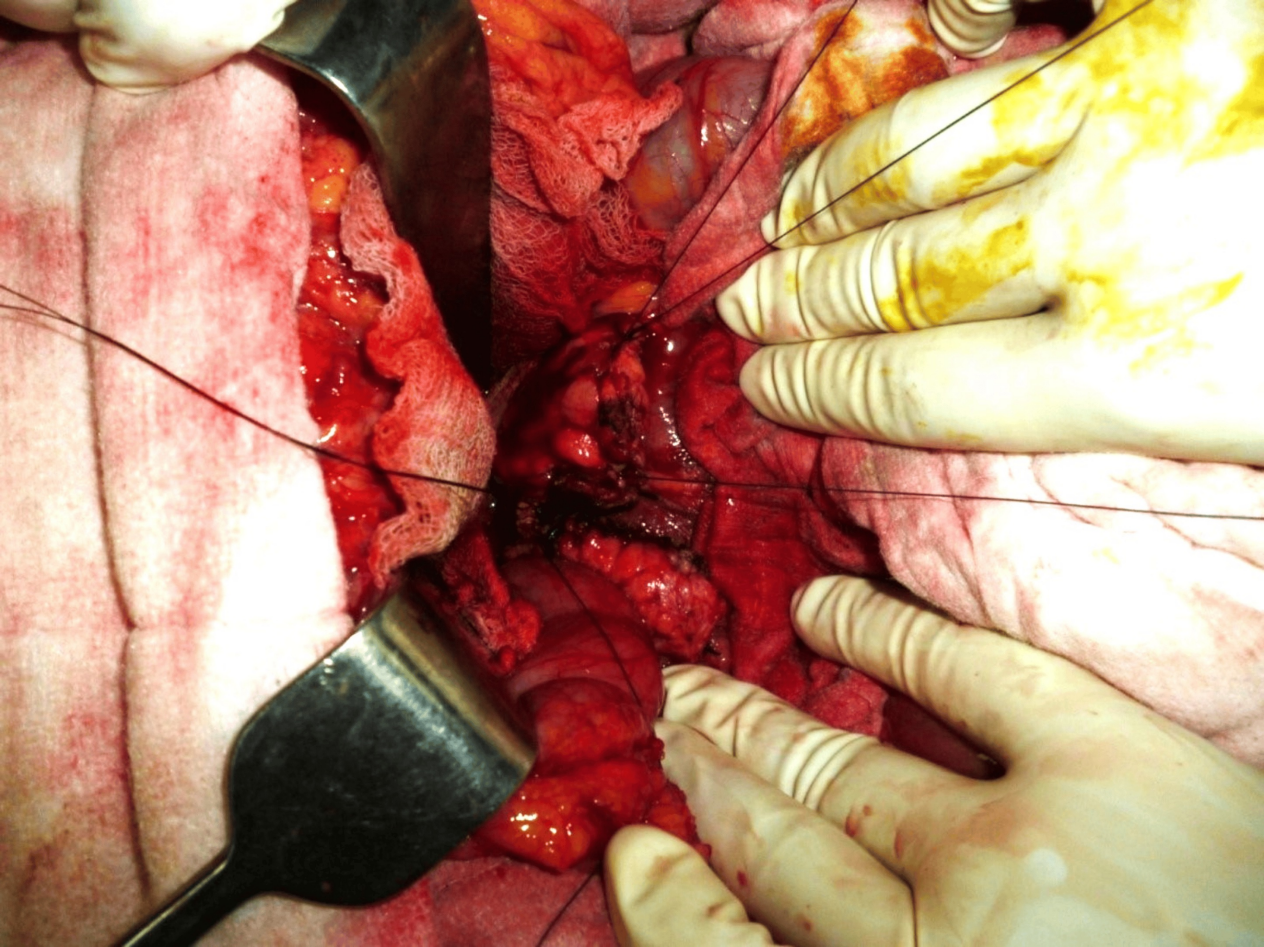
Application of stay sutures with the needle intact.

**Figure 3:**
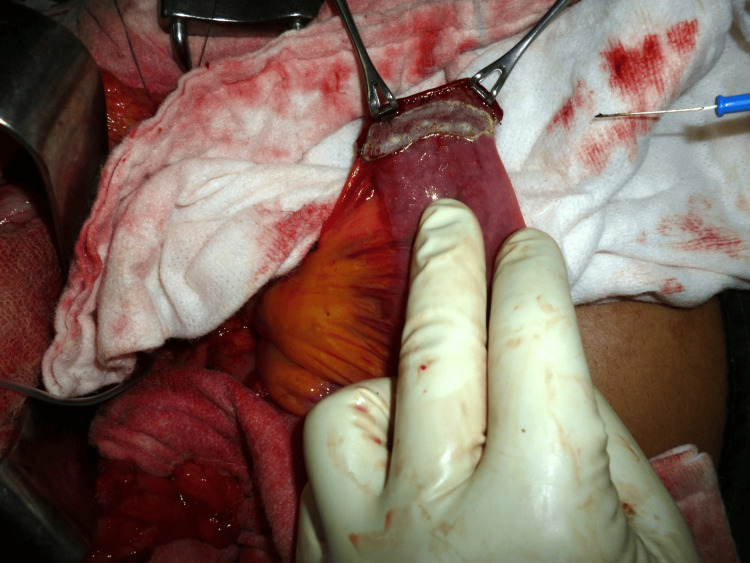
Preparation of the jejunal limb.

**Figure 4:**
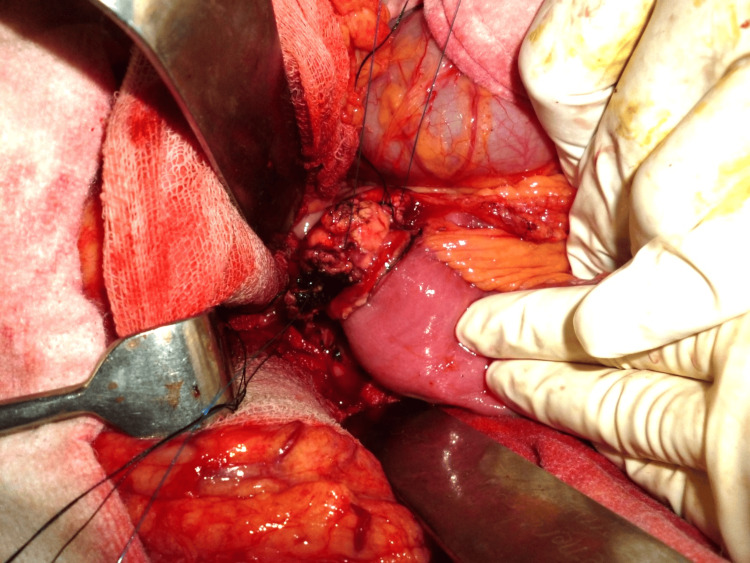
Posterior continuous suture application.

**Figure 5:**
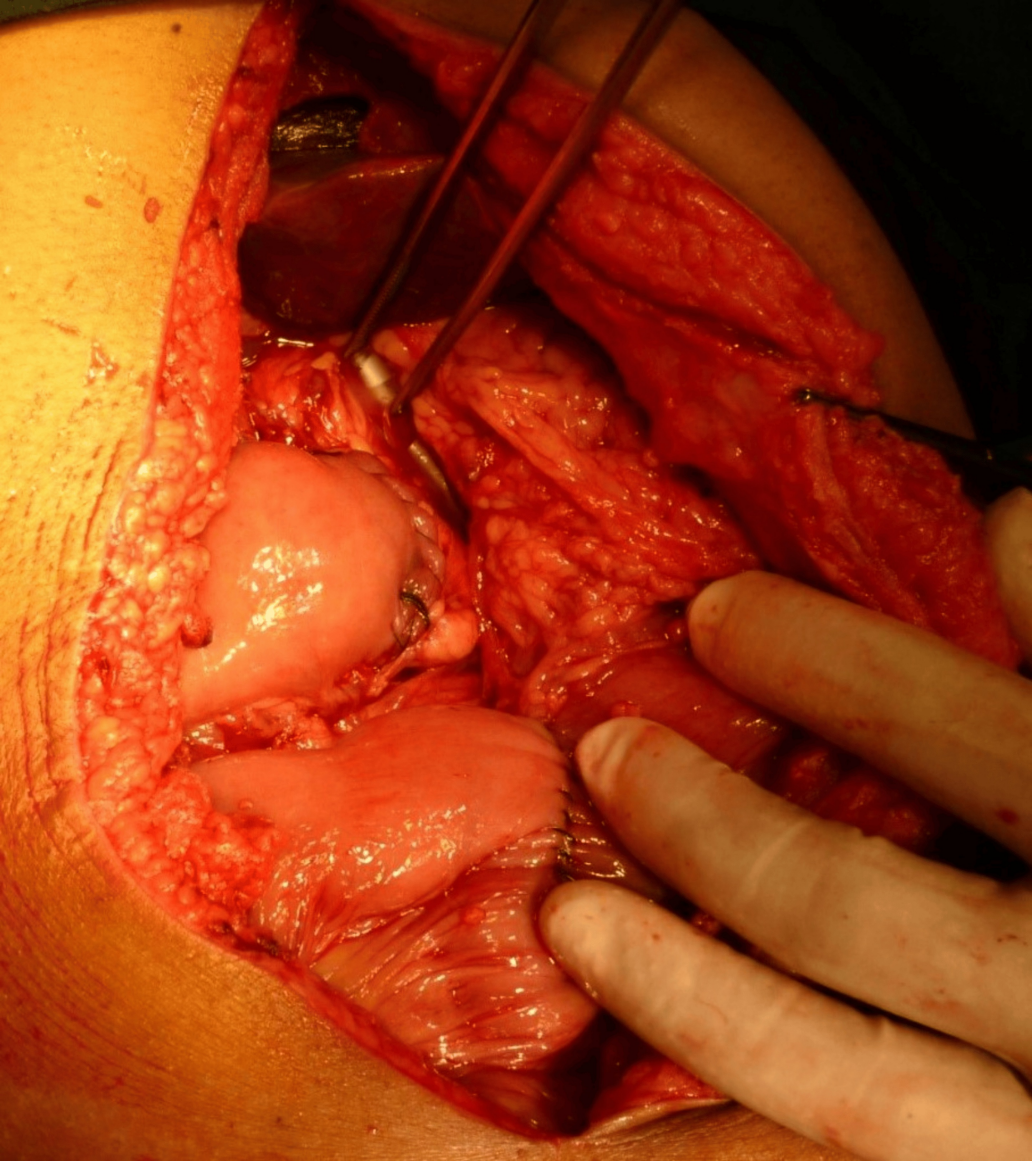
Completed dunking pancreaticojejunal anastomosis.

After transection of the pancreas, the pancreatic stump was mobilized from the retroperitoneum for 4 to 5 cm (Figure 1). Stay sutures with 3-0 silk were made at the superior and inferior border of the pancreatic remnant (Figure 2). The jejunal limb was prepared and the staple line applied during the transection of the jejunum was excised (Figure 3). Using a 3-0 polypropylene suture and starting at the upper border of the pancreas as far distally as mobilized, continuous sutures taking the pancreas capsule on the one side and the serosa of the edge of the jejunum were made to complete the posterior layer of anastomosis (Figure 4). Invagination of the pancreatic stump to the end jejunal limb was done with a previously placed stay suture that was passed through the lumen of the jejunum, followed by the completion of the anterior layer of anastomosis. This procedure invaginates or *dunks* the whole of the cut edges of the pancreas into the jejunal lumen to allow the apposition of the pancreatic capsule to the jejunal mucosa (Figure 5). Hepaticojejunostomy (HJ) was performed 15 cm distal to PJ (continuous with 5-0 Prolene), followed by handsewn antecolic antiperistaltic GJ in two layers with 3-0 Polyglactin (inner layer) and 3-0 Silk (outer layer), 40 cm distal to HJ. Braun’s JJ between afferent and efferent limbs of GJ was done at a distance of 15 cm (single layer, continuous with 3-0 silk).

## Results

**Figure 6:**
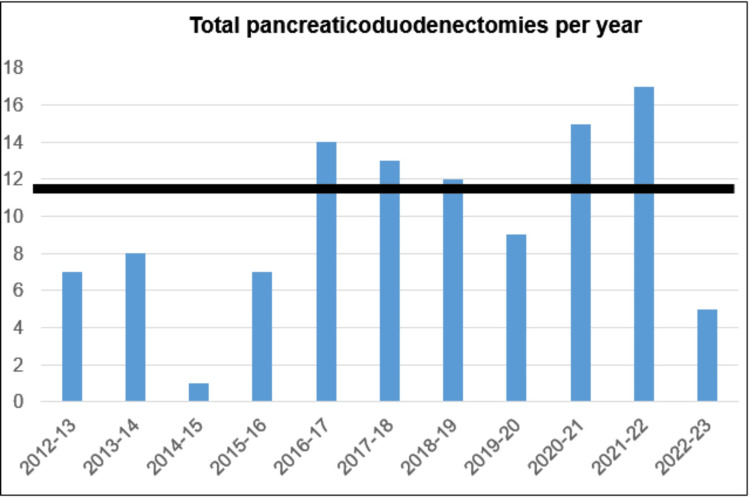
Total pancreaticoduodenectomies per year.

**Figure 7:**
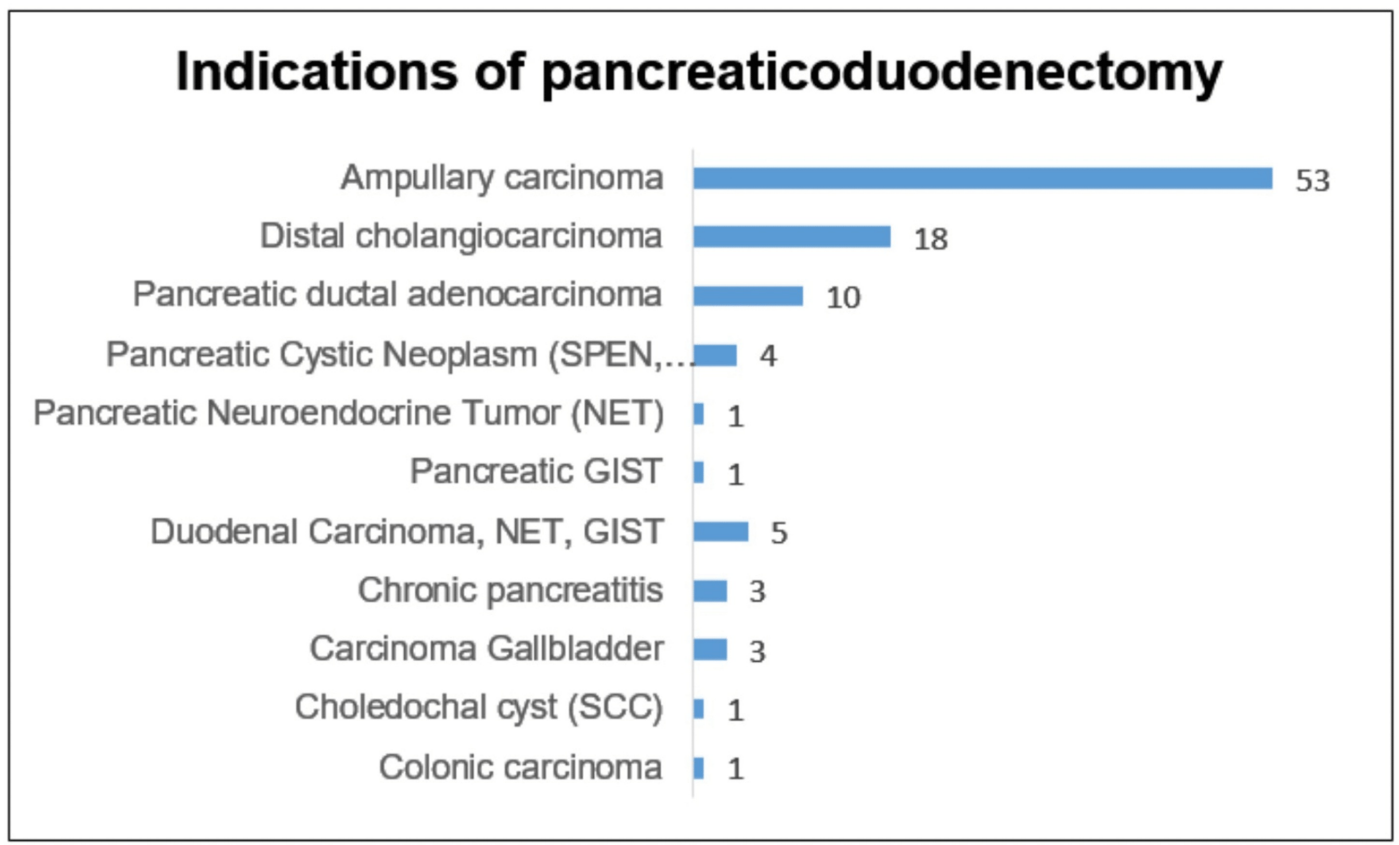
Indications of pancreaticoduodenectomy. GIST: gastrointestinal stromal tumor; SPEN: solid pseudopapillary neoplasm; SCC: squamous cell carcinoma

A total of 108 PDs were performed from October 2012 to February 2023, and 104 were done with the dunking technique. The first 100 were analyzed, with our volume on an increasing trend (Figure 6). The mean age was 50 years with a male predominance. The majority of patients had a preserved performance status. More than half of the patients had ampullary carcinomas (53, 53%), followed by distal cholangiocarcinoma (18, 18%) and pancreatic ductal adenocarcinoma (10, 10%) (Figure 7). In total, 46 (46%) patients required preoperative drainage, and the preferred technique was percutaneous transhepatic biliary drainage (34%). Indications included cholangitis (18%), anticipated delay in surgery due to operating room unavailability (24%), and nutritional conditioning (4%). The average time to surgery following biliary drainage was 14 days (Table 1).

**Table 1:** Baseline characteristics. ASA: American Society of Anesthesiologists; BMI: body mass index; ECOG: Eastern Cooperative Oncology Group; ERCP: endoscopic retrograde cholangiopancreaticography; IQR: interquartile range; PTBD: percutaneous transhepatic biliary drainage; SD: standard deviation

Variables		Frequency (N = 100)
Age (years) (mean ± SD)		50.96 ± 14.97
Sex	Female	36
	Male	64
ASA	1	58
	2	42
ECOG	0	70
	1	25
	2	5
BMI (kg/m^2^) (mean ± SD)		21.28 ± 3.75
Median Bilirubin (µmol/L) (IQR)		85 (25.5–205)
Preoperative biliary drainage		46 (46%)
PTBD		34
ERCP		12
Indications of biliary drainage		
Cholangitis		18
Anticipated delay in surgery		24
Nutritional conditioning		4
Time to surgery after drainage (mean/range)		12 (10–24) days

Soft pancreas was encountered in 52 (52%) patients. The average time for PJ anastomosis was 22 ± 6 minutes. Braun’s anastomosis required only 15 more minutes (Table 2).

**Table 2:** Intraoperative variables. JJ: jejunojejunostomy; PJ: pancreaticojejunostomy; SD: standard deviation

Variables	Frequency (N = 100)
Operative time (hours) (mean/SD)	5.83 ± 1.09
Intraoperative blood loss (mL) (median/SD)	515 ± 194
Pancreatic texture	
Soft	52 (52%)
Firm	48 (48%)
Time for PJ (minutes) (mean/SD)	22 ± 6
Time for JJ (minutes) (mean/SD)	15 ± 3

Clinically relevant POPF (CR-POPF) was observed in 23 (23%) patients. Post-pancreatectomy hemorrhage (PPH) was observed in 21 (21%) patients. DGE was observed in two (2%) patients, and bile leak was observed in four (7%) patients. Seven (7%) patients underwent re-exploration. Overall morbidity rate was 32% with incidence of major complications in 24%. The mortality rate was 11%. Four patients were re-explored for PPH, two for bile leak, and one for POPF, of whom three patients did not survive (Tables 3, 4).

**Table 3:** Procedure-specific complications. DGE: delayed gastric emptying; POPF: postoperative pancreatic fistula; PPH: post-pancreatectomy hemorrhage

Variables	Frequency (N = 100)
POPF	63 (63%)
Grade A	40
Grade B	12
Grade C	11
PPH	21 (21%)
Grade A	4
Grade B	8
Grade C	9
DGE	2
Grade B	2
Bile leak	4 (4%)
Grade A	1
Grade B	2
Grade C	1

**Table 4:** Postoperative variables. CD: Clavien-Dindo; PHLF: post-hepatectomy liver failure; POPF: postoperative pancreatic fistula; PPH: post-pancreatectomy hemorrhage; SD: standard deviation

Variables	Frequency (N = 100)
Length of hospital stay (days) (median/SD)	19.43 ± 7.39
Re exploration	7 (7%)
Bile leak	2
PPH	4
POPF	1
Overall morbidity	32 (32%)
Major complications (CD > IIIA)	24 (24%)
Mortality	11 (11%)
POPF	5
POPF with PPH	3
Bile Leak	1
PHLF	1
Sepsis	1

## Discussion

PD is a potentially curative treatment for carcinomas in the periampullary area, head, or uncinate of the pancreas. POPF is one of the most dreadful complications that can develop following PD. Various studies have reported rates of POPF ranging from 20% to 47% [8,9]. Other debilitating complications such as PPH, intra-abdominal sepsis, and DGE are generally sequelae of POPF. In our study, a POPF rate of 63% is higher and a CR-POPF rate of 23% is consistent with the existing literature.

Risk factors for POPF can be directly related to the pancreas (soft texture, narrow diameter of the pancreatic duct, decreased blood flow, posterior location of the pancreatic duct), high body mass index, preoperative malnutrition, massive intraoperative bleeding, the volume of the pancreatic remnant, surgeon and center experience, and the anastomosis technique [10]. A higher CR-POPF rate in our study could be explained by the soft pancreatic texture encountered in 52% of cases. Posterior mobilization of the pancreatic stump may have contributed to a higher POPF rate.

Dunking PJ and duct-to-mucosa PJ are the most commonly employed techniques of PJ anastomosis. A recent Cochrane review did not show a difference between these techniques on any of the outcomes, including rate of POPF (Grade B or C), postoperative mortality, rate of surgical reintervention, rate of postoperative bleeding, overall rate of surgical complications, and length of hospital stay [11]. A randomized controlled trial from Japan showed that in high-risk patients with a soft pancreas, the dunking technique may reduce the risk of CR-POPF compared with duct-to-mucosa PJ [12]. This finding may be due to the incorporation of both the main pancreatic duct as well as branch ducts into the dunking PJ anastomosis compared to the duct-to-mucosa technique. In our study, the most common indication for PD was ampullary carcinoma where soft pancreatic texture is encountered more commonly.

Braun’s JJ eases the outflow of pancreatic and biliary juice and prevents the stasis of these gastrointestinal juices, providing physical relief for PJ and HJ along with less pressure on the jejunal loop and subsequently on the anastomoses with decreased anastomotic edema and mucosal irritation. Additionally, it potentially stabilizes the gastroenterostomy and prevents kinking, which might reduce limb volvulus. A meta-analysis of PD patients with Braun’s JJ showed lower rates of Grade B and C DGE [7]. In our study, Grade B DGE was encountered in only 2% of patients. Another meta-analysis comparing conventional Child’s reconstruction with Braun’s JJ and isolated PJ showed Braun’s JJ to be associated with a decreased risk for postoperative complications, particularly a decreased risk for clinically relevant DGE, POPF, and bile leaks [13].

Contrary to existing literature, a higher rate of PPH (21%) was observed in our study [14]. There can be multiple explanations for high PPH, including a higher number of soft pancreatic textures leading to higher chances of POPF and eventually PPH. Similarly, the pancreatic stump was exposed to pancreatic juice, which could lead to its degradation and PPH. Moreover, mobilization of the pancreatic stump for reconstruction during dunking PJ could serve as the probable source of PPH.

Our study showed the incidence of CR-POPF with dunking and Braun’s JJ to be comparable to studies published from various parts of the world. However, when we examined PPH, the incidence of PPH was slightly higher than published literature. Thus, the dunking technique is easily adaptable and applicable for all kinds of pancreatic stumps, and with the addition of Braun’s JJ, there is a decrease in the incidence of DGE; hence, selective or routine use can be beneficial.

Limitations of the study are inherent to the retrospective study design, including loss of data, selection bias, and loss to follow-up.

## Conclusions

The dunking technique is versatile, efficient, and applicable to the pancreas of any texture or duct size, although it may elevate the risk of PPH. The addition of Braun’s anastomosis can help minimize DGE, promote early feeding, and reduce complication rates.

## References

[1] Ke Z, Cui J, Hu N (2018). Risk factors for postoperative pancreatic fistula: analysis of 170 consecutive cases of pancreaticoduodenectomy based on the updated ISGPS classification and grading system. Medicine (Baltimore).

[2] Lyu Y, Li T, Cheng Y, Wang B, Chen L, Zhao S (2018). Pancreaticojejunostomy versus pancreaticogastrostomy after pancreaticoduodenectomy: an up-to-date meta-analysis of RCTs applying the ISGPS (2016) criteria. Surg Laparosc Endosc Percutan Tech.

[3] Lyu Y, Wang B, Cheng Y, Xu Y, Du WB (2020). Comparison of surgical outcomes between isolated pancreaticojejunostomy, isolated gastrojejunostomy, and conventional pancreaticojejunostomy after pancreaticoduodenectomy: a systematic review and meta-analysis. BMC Gastroenterol.

[4] Olakowski M, Grudzińska E, Mrowiec S (2020). Pancreaticojejunostomy-a review of modern techniques. Langenbecks Arch Surg.

[5] Sun X, Zhang Q, Zhang J (2016). Meta-analysis of invagination and duct-to-mucosa pancreaticojejunostomy after pancreaticoduodenectomy: an update. Int J Surg.

[6] Zhang S, Lan Z, Zhang J (2017). Duct-to-mucosa versus invagination pancreaticojejunostomy after pancreaticoduodenectomy: a meta-analysis. Oncotarget.

[7] Xu B, Zhu YH, Qian MP, Shen RR, Zheng WY, Zhang YW (2015). Braun enteroenterostomy following pancreaticoduodenectomy: a systematic review and meta-analysis. Medicine (Baltimore).

[8] Callery MP, Pratt WB, Kent TS, Chaikof EL, Vollmer CM Jr (2013). A prospectively validated clinical risk score accurately predicts pancreatic fistula after pancreatoduodenectomy. J Am Coll Surg.

[9] Hayashi H, Amaya K, Fujiwara Y (2021). Comparison of three fistula risk scores after pancreatoduodenectomy: a single-institution retrospective study. Asian J Surg.

[10] Bassi C, Marchegiani G, Dervenis C (2017). The 2016 update of the International Study Group (ISGPS) definition and grading of postoperative pancreatic fistula: 11 years after. Surgery.

[11] Hai H, Li Z, Zhang Z, Cheng Y, Liu Z, Gong J, Deng Y (2022). Duct-to-mucosa versus other types of pancreaticojejunostomy for the prevention of postoperative pancreatic fistula following pancreaticoduodenectomy. Cochrane Database Syst Rev.

[12] Senda Y, Shimizu Y, Natsume S (2018). Randomized clinical trial of duct-to-mucosa versus invagination pancreaticojejunostomy after pancreatoduodenectomy. Br J Surg.

[13] Schorn S, Demir IE, Vogel T (2019). Mortality and postoperative complications after different types of surgical reconstruction following pancreaticoduodenectomy-a systematic review with meta-analysis. Langenbecks Arch Surg.

[14] Maccabe TA, Robertson HF, Skipworth J, Rees J, Roberts K, Pathak S (2022). A systematic review of post-pancreatectomy haemorrhage management stratified according to ISGPS grading. HPB (Oxford).

